# Palmoplantar-predominant bullous pemphigoid with acral purpuric lesions: An unusual presentation

**DOI:** 10.1016/j.jdcr.2025.11.033

**Published:** 2025-12-01

**Authors:** Lucía Martínez Rozas, María Uxúa Floristán Muruzábal, Fernando Javier Pinedo-Moraleda, José Luis López Estebaranz

**Affiliations:** a Department of Dermatology, Resident in Dermatology in Fundación Alcorcón Hospital, Madrid, Spain; b Department of Dermatology, Consultant Dermatologist in Fundación Alcorcón Hospital, Madrid, Spain; c Department of Anatomical Pathology, Consultant Pathologist in Fundación Alcorcón Hospital, Madrid, Spain

**Keywords:** acral involvement, autoimmune blistering disease, bullous pemphigoid, dipeptidyl peptidase-4 inhibitors, purpuric, tissue factor

An 81-year-old man with hypertension, type 2 diabetes mellitus, dyslipidemia, and a prior ischemic stroke presented with a 2-month history of tense, pruritic, clear, and hemorrhagic bullae predominantly on the palms and soles, with few lesions on the trunk. His long-term medications included insulin, sitagliptin, metformin, furosemide, acetylsalicylic acid, atorvastatin, olmesartan, amlodipine, hydrochlorothiazide, and carbidopa-levodopa.

A punch biopsy from a fresh blister revealed a subepidermal split with a perivascular eosinophil-rich infiltrate. Direct immunofluorescence demonstrated a linear deposition of C3 along the basement membrane zone, and ELISA testing showed positivity for anti-BP180 and anti-BP230 antibodies. These findings confirmed the diagnosis of bullous pemphigoid (BP) (Fig 1, Fig 2, Fig 3).

**Fig 1:**
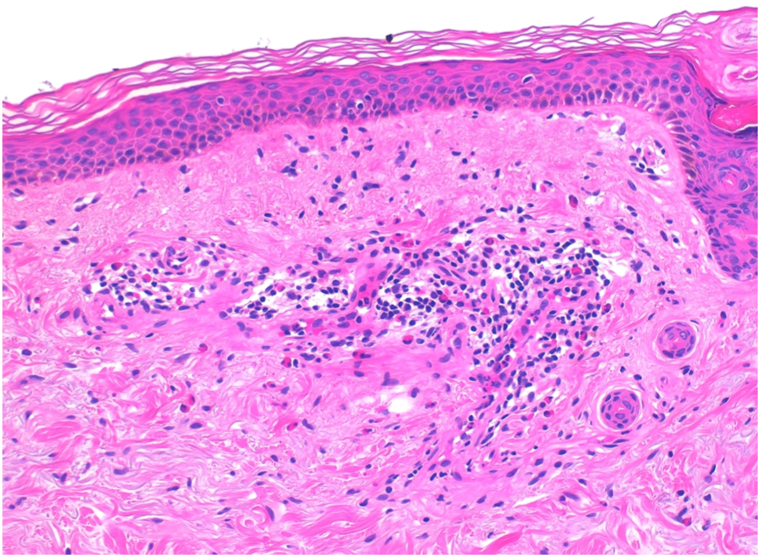
Histological examination revealed a perivascular and interstitial infiltrate with a predominance of eosinophils, without evidence of a well-formed bullous lesion.

**Fig 2:**
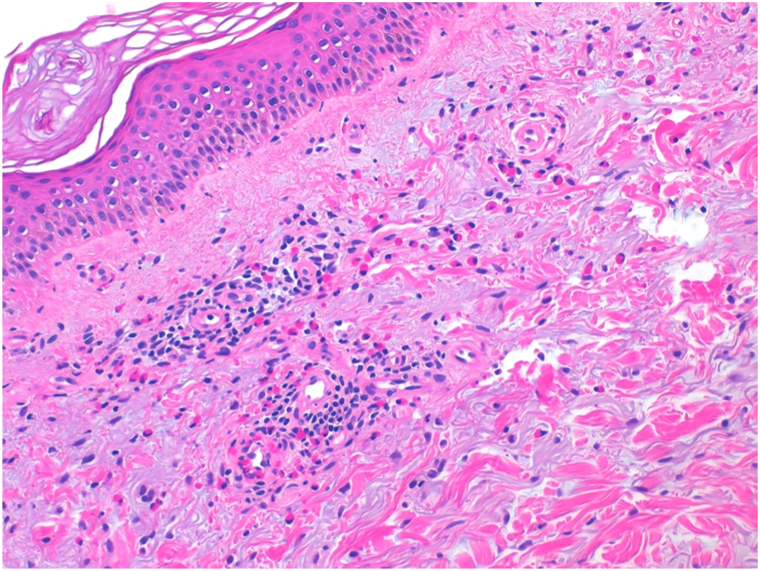
Histological examination revealed a perivascular and interstitial infiltrate with a predominance of eosinophils, without evidence of a well-formed bullous lesion.

**Fig 3:**
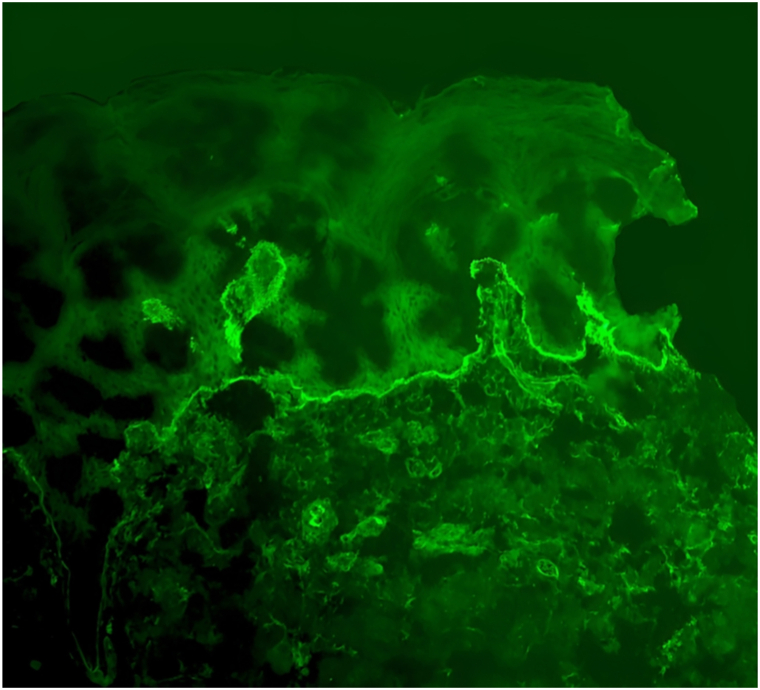
Direct immunofluorescence demonstrated a mild linear deposition of C3 (+/+++) along the dermoepidermal junction.

Sitagliptin, a DPP-4 inhibitor associated with drug-induced BP, was discontinued. Topical clobetasol and oral antihistamines were initiated, achieving marked improvement. Only a brief oral prednisone taper (30 mg) was required for pruritus control. At follow-up, the patient remained clinically stable with occasional isolated acral lesions, mainly purpuric in appearance.

**Question: Which of the following best explains the presence of purpuric acral lesions in this patient with bullous pemphigoid?**

**A.** Concomitant dermatitis herpetiformis
**B.** Drug-induced vasculitis due to sitagliptin
**C.** Localized mechanical trauma on acral skin
**D.** Tissue factor–mediated eosinophilic inflammation and microvascular thrombosis
**E.** Secondary bacterial infection of bullae

Correct answer: D.

## Discussion

**Fig 4:**
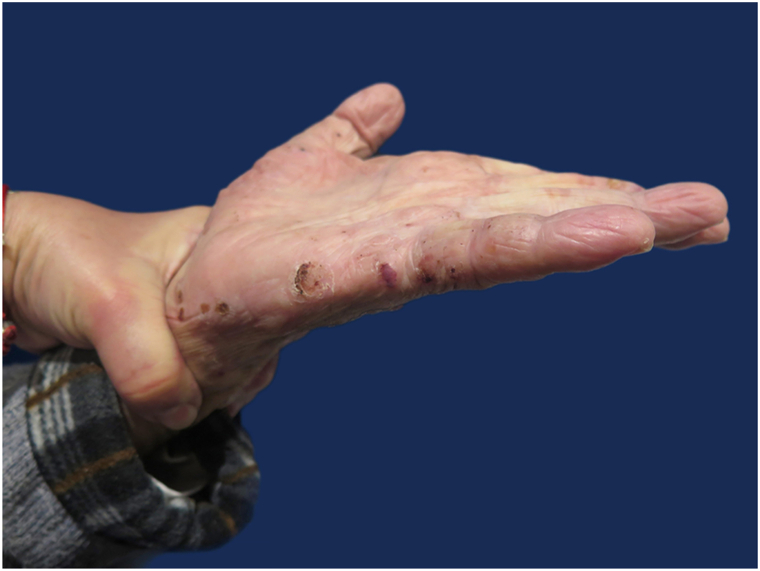
Clinically, the patient presented with tense bullae and vesicles filled with clear and hemorrhagic fluid, predominantly distributed acrally, involving the palms.

**Fig 5:**
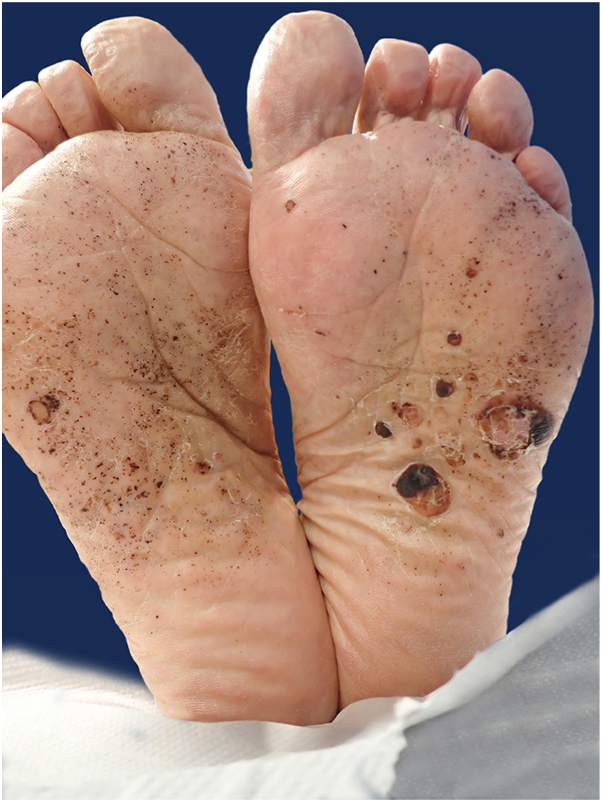
Clinically, the patient presented with tense bullae and vesicles filled with clear and hemorrhagic fluid, predominantly distributed acrally, involving the soles.

BP is the most frequent autoimmune blistering dermatosis of the elderly.^1^ It is caused by autoantibodies directed against hemidesmosomal proteins BP180 and BP230, leading to dermoepidermal separation. When limited to the palms and soles (in less than 5% of cases), bullous pemphigoid may mimic pompholyx or contact dermatitis, which can delay diagnosis (Figs 4 and 5).

Purpuric acral lesions in BP have not been described as an independent clinical variant; however, 2 similar cases to ours have been previously reported.^2^^,^^3^ Other conditions that may present with vesicles or hemorrhagic papules on acral areas include dermatitis herpetiformis and the acral hemorrhagic variant of Darier disease. In such settings, direct immunofluorescence and serologic studies are essential to distinguish BP from these mimickers.

Recent research highlights the role of tissue factor in the pathogenesis of BP.^4^ Eosinophils, abundant in lesional skin, are a major intravascular source of tissue factor, which activates the extrinsic coagulation cascade and enhances leukocyte recruitment. Overexpression of tissue factor correlates with eosinophilia and disease activity,^5^ potentially producing localized microvascular thrombosis and capillary damage. This mechanism explains the purpuric lesions observed in our patient.

## Teaching points

• Bullous pemphigoid should be considered in purpuric and vesiculobullous eruptions confined to the palms and soles in elderly patients.
• Purpuric lesions in BP, though rare, may result from eosinophil-induced coagulation activation.
• Sitagliptin and other DPP-4 inhibitors are recognized BP triggers and should be discontinued when disease appears.

## Conflicts of interest

None disclosed.
